# Role of STAT3 and NRF2 in Tumors: Potential Targets for Antitumor Therapy

**DOI:** 10.3390/molecules27248768

**Published:** 2022-12-10

**Authors:** Yanjun Tian, Haiqing Liu, Mengwei Wang, Ruihao Wang, Guandong Yi, Meng Zhang, Ruijiao Chen

**Affiliations:** 1 Medical Laboratory of Jining Medical University, Jining Medical University, Jining 272067, China; 2 Department of Physiology, School of Basic Medical Sciences (Institute of Basic Medical Sciences), Shandong First Medical University & Shandong Academy of Medical Sciences, Jinan 250024, China; 3 School of Stomatology, Jining Medical University, Jining 272067, China; 4 School of Mental Health, Jining Medical University, Jining 272067, China; 5 School of Nursing, Jining Medical University, Jining 272067, China

**Keywords:** signal transduction, STAT3, NRF2, tumor, antitumor therapy

## Abstract

Signal transducer and activator of transcription 3 (STAT3) and nuclear factor erythroid-derived 2-like 2 (NRF2, also known as NFE2L2), are two of the most complicated transcription regulators, which participate in a variety of physiological processes. Numerous studies have shown that they are overactivated in multiple types of tumors. Interestingly, STAT3 and NRF2 can also interact with each other to regulate tumor progression. Hence, these two important transcription factors are considered key targets for developing a new class of antitumor drugs. This review summarizes the pivotal roles of the two transcription regulators and their interactions in the tumor microenvironment to identify potential antitumor drug targets and, ultimately, improve patients’ health and survival.

## 1. Introduction

Signal transducer and activator of transcription 3 (STAT3) belongs to the STAT family, which includes STAT1, STAT2, STAT3, STAT4, STAT5a, STAT5b, and STAT6, and mediates signal transduction from the cell membrane to the nucleus in multiple intracellular and extracellular activities [1,2]. As a crucial transcription factor, STAT3 exerts a vital role on all STAT proteins. As it is essential for early development, it gets involved in regulating the transcription of a good many crucial genes related to cell proliferation, differentiation, apoptosis, survival, angiogenesis, inflammation, immunity, and metastasis, thereby participating in various physiological and pathological processes [3,4,5]. There is mounting evidence showing that STAT3 plays a crucial role in various diseases, such as cancer [6,7,8], cerebrovascular diseases [9,10], cardiovascular diseases [11,12], and obesity [13].

**Figure 1:**
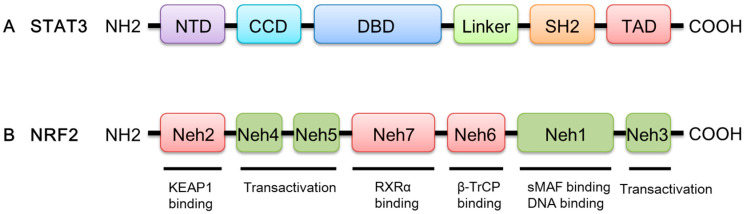
A schematic diagram of the domain structures of STAT3 and NRF2. (**A**) STAT3 has six functional domains, including NTD, CCD, DBD, Linker, SH2, and TAD. (**B**) NRF2 has seven conserved domains labeled Neh1–Neh7.

The *STAT3* gene is located on chromosome 17q21 [14,15]. The STAT3 protein consists of 770 amino acids and has six conserved domains (Figure 1). The amino-terminal domain (NTD) of STAT3 performs multiple functions, including protein–protein interactions, cooperative DNA binding, and nuclear translocation [16]. STAT3 interacts with other transcription factors and regulatory proteins via coiled-coil domain (CCD) [17]. The DNA-binding domain (DBD) facilitates STAT3 interactions with target genes. STAT3 dimerization is formed via the Src homology-2 (SH2) domain by identifying phosphorylated Tyr-705 of another STAT monomer [17]. The phosphorylation of serine sites on the C-terminal transcription activation domain (TAD) promotes the assembly of STAT3 with other transcriptional activators [17,18,19]. The structure of the STAT3 protein determines its special functions, which lays the foundation for signal transduction.

Nuclear factor erythroid-derived 2-like 2 (NRF2) possesses a unique Cap ‘n’ Collar (CNC) motif followed by a basic leucine zipper (bZip), which belongs to the CNC transcription factor family. Human NRF2 contains seven highly conserved domains, namely NRF2-ECH (erythroid cell-derived protein with CNC homology) homology and (Neh) 1–Neh7; each domain has its own unique function (Figure 1). A bZip DNA-binding domain and a heterodimerization domain together form the Neh1 domain, which enables DNA-binding to the antioxidant response element (ARE) and the dimerization of NRF2 with small musculoaponeurotic fibrosarcoma (sMAF) proteins [20]. Neh2 is a negative regulatory domain of NRF2, which is crucial for Kelch-like ECH-associated protein 1 (KEAP1)-mediated repression of NRF2 [21]. The C-terminal Neh3 domain acting in parallel with the Neh4 and Neh5 domains has a transactivation-like activity to activate the transcription of NRF2 target genes [22,23]. The Neh6 domain includes two binding sites for β-transducin repeat-containing protein (β-TrCP), DSGIS, and DSAPGS, leading to glycogen synthase kinase 3 beta (GSK3β)-mediated NRF2 degradation in a KEAP1-independent manner [24,25]. Finally, Neh7 contains a domain that mediates a direct interaction between NRF2 and the DBD of retinoid X receptor α (RXRα), which suppresses the transcriptional activity of NRF2 by inhibiting the recruitment of coactivators to Neh4 and Neh5 domains [26].

Numerous studies indicate that NRF2 has a vital role in regulating redox and metabolic homeostasis by inducing corresponding target genes [27,28,29]. Due to the post-translational regulation by the ubiquitin-proteasome system (UPS), the cellular level of NRF2 is normally very low. However, when an organism is exposed to endogenous and environmental stresses, the action of the UPS is blocked, leading to the activation of NRF2 signaling. The newly translated NRF2 translocates to the nucleus, binds to sMAF proteins, and transcribes ARE-regulated genes. Multiple research data have confirmed that NRF2-deficient mice are more vulnerable to the toxicity and carcinogenesis of various xenobiotic stresses [30,31,32,33]. Many compounds, including natural products and others, have been found to activate NRF2 to protect cells from damage [34]. However, just as NRF2 can safeguard normal cells against insult, it can also protect tumor cells from damage, facilitating their transformation, growth, metastasis, and chemoresistance. Therefore, a growing number of researchers are committed to the discovery and development of NRF2 inhibitors.

Numerous studies have reported that STAT3 and NRF2 are hyperactive in tumors, with important and intricate regulatory functions. STAT3 and NRF2 interact to regulate tumor progression [35,36]. Depending on the situation, they can either prevent or promote cancer progression [35,36]. This review focuses on the crucial roles of two important transcription regulators (i.e., STAT3 and NRF2) and their interactions in the tumor microenvironment to identify potential antitumor drug targets and ultimately improve the health and survival of cancer patients.

## 2. Role of STAT3 Signaling

### 2.1. STAT3 Signal Transduction Cascade

Many factors including receptor tyrosine kinases (RTK), Janus kinases (JAKs), cytokines, and some non-receptor tyrosine kinases such as Src and Abl can induce the phosphorylation of tyrosine (705) and serine (727) residues of STAT3, which will activate the STAT3 signal transduction cascade [4,6,37,38,39,40]. Upon activation, STAT3 dissociates from the receptor/kinase complex to form homodimers or heterodimers via the SH2 domain. This is followed by nuclear translocation, DNA binding, and activation of target genes, including pro-proliferative/anti-apoptotic genes, angiogenic genes, metastatic genes, and the STAT3 gene itself [41]. Additionally, it is reported that unphosphorylated STAT3 (u-STAT3) can drive the expression of many genes, such as *interleukin-6* (*IL-6*), *IL-8*, *C-C motif chemokine ligand 5* (*CCL5*), *regulated upon activation*, *normal T-cell expressed and secreted* (*RANTES*), *mesenchymal-epithelial transition* (*MET*), and *muscle RAS oncogene homolog* (*MRAS*), via a non-canonical pathway independent of phosphorylation [42]. Therefore, phosphorylation is not necessary for STAT3 activation, and STAT3 regulates corresponding target genes through different methods.

STAT3 is tightly negatively regulated in unstimulated cells by a number of modulators, including the protein inhibitor of activated STAT3 (PIAS3), suppressor of cytokine signaling (SOCS) proteins, protein tyrosine phosphatases (PTPs), and ubiquitin enzymes, which can suppress the expression and nuclear translocation of STAT3 [43]. For example, PIAS3 expression negatively correlates with STAT3 signal transduction in cervical cancer (CC) cells, probably by repressing the DNA binding activity of STAT3 [44,45,46]. Baek et al. discovered that resveratrol can induce SOCS-1 expression, suppress STAT3 phosphorylation, and restrain proliferation, which thereby inhibits the STAT3 signaling pathway in squamous cell carcinoma of the head and neck (SCCHN) [47]. Numerous studies have shown that STAT3 signaling can be inhibited by various PTPs, such as protein tyrosine phosphatase non-receptor type 6 (PTPN6, also known as SHP1), protein tyrosine phosphatase non-receptor type 11 (PTPN11, also known as SHP2), CD45, protein tyrosine phosphatase non-receptor type 1 (PTPN1, also known as PTP1B), protein tyrosine phosphatase non-receptor type 2 (PTPN2, also known as TC-PTP), and phosphatase and tensin homologue deleted on chromosome 10 (PTEN) [48,49,50,51,52]. Furthermore, Nie et al. demonstrated that paeoniflorin inhibited proliferation and induced apoptosis in human glioma cells via ubiquitin–proteasome pathway (UPP)-mediated STAT3 degradation [53].

STAT3 activation and inactivation are highly regulated in normal cells, whereas in tumor cells, downregulation of the endogenous negative regulators of the STAT3 signaling pathway leads to enhanced proliferation and malignancy [54,55].

### 2.2. STAT3 in Tumor Cells

**Figure 2:**
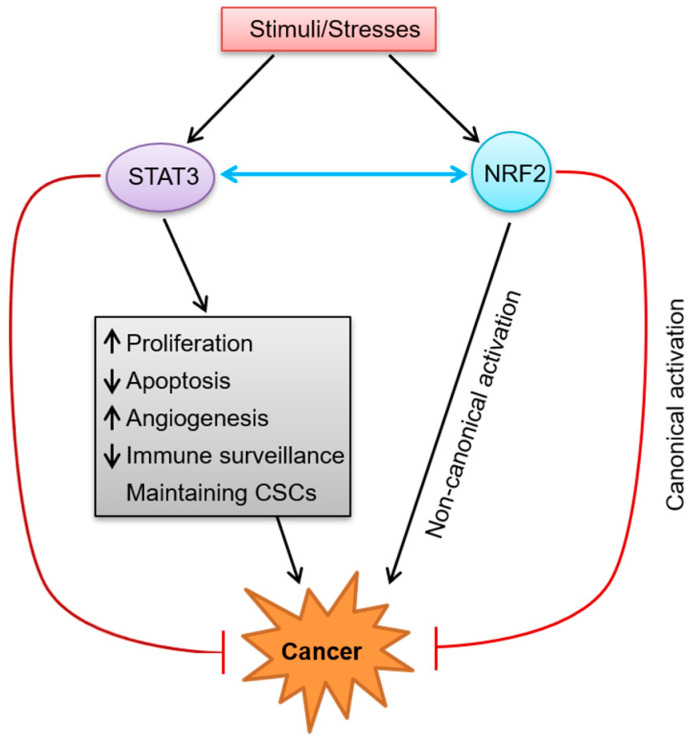
The dual roles of STAT3 and NRF2 in cancer and their crosstalk. When cells are stimulated by various kinds of stimuli/stresses, STAT3 and NRF2 will be activated. Activated STAT3 promotes cancer development through multiple mechanisms including promoting cell proliferation, suppressing apoptosis, facilitating angiogenesis, escaping immune surveillance, and maintaining CSCs. On the other hand, activated STAT3 has anti-tumor function. Just as STAT3, NRF2 can not only promote carcinogenesis via a non-canonical activation mode, but also inhibit the development of cancer through a canonical mode. In addition, STAT3 and NRF2 can also interact with each other. (The black arrow represents activation, the red arrow represents inhibition, and the blue double-head arrow represents interaction/crosstalk.)

The activity of STAT3 is required for embryonic development, but can also lead to tumorigenesis and tumor progression, making it a double-edged sword (Figure 2).

#### 2.2.1. Functions of STAT3 in Cell Proliferation and Survival in Tumors

A growing body of research data indicates that sustained STAT3 activation is necessary for abnormal cell proliferation and survival during tumorigenesis, whereas blocking STAT3 signaling inhibits cell proliferation and promotes apoptosis in multiple cancers. You et al. found that IL-26 could promote proliferation and suppress apoptosis in human gastric cancer cells by increasing the expression of Bcl-2, Bcl-xL, and c-Myc, which are associated with STAT3 activation [56], whereas Kanai et al. found that differentiation-inducing factor-1 (DIF-1) suppressed gastric cancer cell proliferation by inhibiting STAT3 activity in a MEK/ERK-dependent manner [57]. LL1 can also induce apoptosis and inhibit metastasis in colorectal cancer cells by selectively blocking STAT3 activation [58]. In addition to the upregulation of STAT3 activity, the proliferation and survival of tumor cells also involve the downregulation of wild-type p53. Furthermore, STAT3 blockade in cancer cells can upregulate p53, leading to p53-dependent tumor cell apoptosis and UV-induced tumor cell growth arrest [59].

#### 2.2.2. Contribution of STAT3 to Tumor Angiogenesis

Angiogenesis is considered to be a key step for tumor growth and metastasis. STAT3 can participate in angiogenesis by interacting with various growth factors including vascular endothelial growth factor (VEGF), basic fibroblast growth factor (bFGF), and platelet-derived growth factor (PDGF), leading to the degradation of vascular basement membrane, proliferation and migration of vascular epithelial cells, and reconstruction and dissolution of new blood vessels [60,61]. In addition, STAT3 can induce hypoxia-inducible factor-1α (HIF1α), another key regulator of angiogenesis, in the tumor microenvironment [62,63,64]. Notably, several studies have shown that matrix metalloproteinases (MMPs) such as MMP2 and MMP9 contribute to tumor angiogenesis, which can be suppressed by inhibiting STAT3 activity [64,65].

#### 2.2.3. STAT3 in Immune System Evasion

The immune system exerts a pivotal function in cancer prevention by detecting and removing abnormal transformed cells that rely on some immune cells. First of all, Innate immune cells including macrophages, natural killer (NK) cells, and dendritic cells (DCs), as well as adaptive immune cells (T helper cell type 1 (Th1)), can destroy tumor cells through a variety of mechanisms [66]. Nevertheless, abnormal cells can escape immune surveillance and ultimately result in malignant tumors via tumor-associated macrophages (TAMs) and myeloid-derived suppressor cells (MDSCs) through complex mechanisms. These include decreased expression of cancer antigens and major histocompatibility complex (MHC)-I and MHC-II molecules on T cells, and increased angiogenetic, metastatic, and growth factors or immunosuppressive cytokines [4,67,68,69,70].

Increasing evidence suggests that STAT3 is involved in regulating tumor cell immune evasion. Wang et al. indicated that in tumors, activating STAT3 can negatively regulate the inflammatory cytokines’ expression and inhibit DC maturation, resulting in a decrease of MHC-II expression, antigen presentation, and T-cell immunity [71]. Numerous studies show that, within the tumor microenvironment, STAT3 signaling induces pro-carcinogenic cytokines (such as IL-6, IL-10, and IL-23) and inhibits anti-carcinogenic cytokines (e.g., IL-12), thereby promoting tumor immune evasion and cancer progression [72]. For instance, Kortylewski et al. found that STAT3 facilitated IL-23-mediated pro-carcinogenic immune responses and restrained IL-12-dependent antitumor immunity [73]. In addition, STAT3 inhibits the expression of CXC-chemokine ligand 10 (CXCL10), which can significantly enhance NK cell cytotoxicity against tumor cells [74]. STAT3 activation can induce cancer-promoting inflammation mediated by nuclear factor-kappa B (NF-κB) and IL-6/GP130/JAK pathways while suppressing NF-κB- and STAT1-mediated Th1 antitumor immune responses by decreasing the expression of antitumor cytokines such as IL-12 and IFN [73,75,76].

#### 2.2.4. Function of STAT3 in Cancer Stem Cells

Many tumors contain a subpopulation of cells that possess the same properties as stem cells in normal tissue, known as cancer stem cells (CSCs) or cancer stem-like cells [77,78,79,80]. CSCs are capable of self-renewal and can generate a wide variety of tumor cells, thereby facilitating tumor heterogeneity. Moreover, CSCs cause tumor recurrence, metastasis, and drug resistance. Remarkably, STAT3 exerts a vital role in promoting cancer through regulating the activities of CSCs. STAT3 can maintain the population of CSCs and their “stem-like” characteristics through various intricate mechanisms. Firstly, STAT3 plays an essential role in maintaining the expression of CSC marker genes such as *CD24*, *CD34*, *CD38*, *CD44*, *CD90*, and *CD133*, which are essential for the stem cell phenotype. The evidence suggests that the function of STAT3 in CSCs is achieved via crosstalk between activated STAT3 and the marker genes of pluripotent embryonic stem (ES) cells, such as *OCT3/4* and *NANOG* [81,82,83,84]. Secondly, STAT3 is involved in epithelial–mesenchymal transition (EMT)-related pathways; this is one of the chief accepted mechanisms for CSC formation [85,86]. Furthermore, STAT3 participates in the expression and protein stability of HIF-1α, and either regulates or is regulated by VEGF, which plays an essential role in maintaining CSCs’ self-renewal [87,88]. STAT3 can also protect CSCs from the innate immune system, as inhibiting activated STAT3 can reverse the suppression of phagocytosis and the secretion of IL-10 in glioma CSCs (gCSCs) [89]. Moreover, STAT3 feedback activation might perform an important role in mediating drug resistance to a wide range of targeted cancer therapies and chemotherapies [90].

Considering the prominent functions of STAT3 in maintaining the characteristics of CSCs, it is reasonable to speculate that STAT3 inhibition can markedly or permanently eliminate CSCs for achieving cancer prevention. One study found that STAT3 can be selectively inhibited by the chemical compound stattic or by siRNA, which can abolish CSC proliferation [91]. In another study, researchers found that BBI608, a small molecule STAT3 inhibitor known to inhibit cancer recurrence, progression, and metastasis, could suppress the expression of stemness-associated genes, deplete ALDH1-positive CSCs, and overcome cisplatin resistance in non-small cell lung cancer (NSCLC) [92].

### 2.3. Dual Roles of STAT3 in Cancer

In addition to the tumor-promoting effects described previously, plenty of evidence indicates that STAT3 can be used as a tumor suppressor in various tumors under certain conditions. For instance, in glial cells, STAT3 exerts a tumor-suppressive effect with complete PTEN function, whereas in epidermal growth factor receptor (EGFR)vIII-positive tumors, it plays an oncogenic role, both of which are mediated by different signaling pathways [93,94]. In a normal cell with intact PTEN function, the protein kinase B (Akt, also known as PKB) is inhibited, allowing forkhead box O3 (FOXO3) to activate transcription of the leukemia inhibitory factor receptor β (LIFRβ) gene. STAT3 is then activated by phosphorylation and represses transcription of the IL8 gene, which ultimately suppresses glioma cell proliferation and invasiveness [93,94]. However, in human glioblastoma cells, PTEN loss leads to the downregulation of LIFRβ expression via Akt inhibition of FOXO3, STAT3 is no longer active, and its inhibition of IL8 gene is removed, leading to upregulation of IL8, which drives malignant glial transformation [93,94]. In colorectal cancer, the tumor inhibitory effect of STAT3 is achieved by suppressing the expression of Snail-1 by promoting GSK3β activity [95]. The action of STAT3 in lung cancer is also ambivalent. In lung adenocarcinomas, STAT3 can be activated by mutant EGFR via driving the expression of the IL-6 cytokine, which activates the gp130/JAK signaling pathway, while blocking this pathway will repress cell-cycle progression, cell growth, and tumorigenesis [96]. However, in Kirsten rat sarcoma and viral oncogene (KRAS)-mutant lung adenocarcinoma, STAT3 exerts an unexpected tumor-suppressive effect by sequestering NF-κB in the cytoplasm, thus decreasing IL-8 expression induced by NF-κB [97]. Specifically, genetic ablation of *Stat3* in murine as well as *STAT3* in human cells leads to an increase of NF-κB-induced expression of CXCL1/IL-8, which contributes to infiltration of myeloid cells as well as vascularization; while inhibiting CXCL1’s cognate receptor, CXCR2 can normalize tumor vascularization and microenvironment and reduce tumor burden [97]. Considering the aforementioned roles of STAT3, we must consider the dual effects of the STAT3 signaling pathway when using it as a drug target. The therapeutic purpose can be achieved by seeking advantages and avoiding disadvantages.

## 3. Intricacies of NRF2 Regulation in the Tumor Microenvironment

### 3.1. NRF2 Signaling Pathway

Under normal conditions, NRF2 is negatively regulated by three E3 ubiquitin ligase complexes: the KEAP1-cullin 3 (CUL3)-ring box 1 (RBX1) complex, the β-TrCP-S-phase kinase-associated protein 1 (SKP1)-CUL1-RBX1 complex, and the Hmg-CoA reductase degradation protein 1 (HRD1) [29,98,99,100]. However, when the organism is exposed to endogenous and environmental stresses, NRF2 degradation is interrupted by the inhibition of the UPS, and newly translated NRF2 translocates to the nucleus, binds to sMAF proteins, and transcribes ARE-driven genes. The extensive cytoprotective genes regulated by NRF2 are crucial for suppressing the oxidative, proteotoxic, and metabolic stresses which facilitate malignant transformation. The transient activation of NRF2 during stress is beneficial to health, while a sustained activation of NRF2 has detrimental effects.

#### 3.1.1. Canonical Activation of NRF2

A large body of evidence shows that KEAP1 has become a crucial regulator of the NRF2-mediated signaling pathway. It is generally believed that KEAP1 can function as a molecular switch to sense the imbalance in redox homeostasis and turn on or off the NRF2 signaling pathway [101,102]. Under general conditions, the activity of NRF2 is negatively regulated by KEAP1. During stress, NRF2 upregulation can be induced by oxidative or electrophilic modification of KEAP1 cysteines (i.e., Cys151), which suppresses the formation of the NRF2–KEAP1 complex and results in diminished NRF2 ubiquitination, thereby initiating the canonical NRF2 signaling pathway [103,104,105,106]. Thus, newly translated NRF2 translocates to the nucleus, binds to sMAF proteins, and transcribes ARE-regulated cytoprotective target genes to maintain redox homeostasis [20]. When redox homeostasis is restored, KEAP1 travels into the nucleus to dissociate NRF2 from the ARE and returns NRF2 to the cytosol for ubiquitination and degradation to inhibit the sustained activation of NRF2 [107]. This pattern of NRF2-activation regulation is an immediate consequence of oxidative or electrophilic stresses and is referred to as “canonical activation”. In terms of chemoprevention, NRF2 has been shown to be activated via the canonical mode by various dietary compounds or synthetic chemicals [108]. The treatment strategies for many diseases, including cancer, are based on utilizing the protective capacity of the NRF2 response, which is achieved by transient NRF2 activation via oxidative or electrophilic modification of KEAP1 [109,110,111,112]. Therefore, the canonical activation of NRF2 is crucial to switch on the detoxification of harmful carcinogens and relieve excessive stress to avoid malignant transformation (Figure 2).

#### 3.1.2. Non-Canonical Activation of NRF2

Another important pathway during stress is the autophagy-lysosome pathway, a highly regulated cellular degradation pathway which is responsible for removing damaged, degenerative, and aging proteins and organelles, such as oxidatively damaged proteins and dysfunctional mitochondria. Autophagy dysfunction leads to the accumulation of pathogenic proteins and organelles, which is the root cause of many diseases, including metabolic disorders, neurodegenerative diseases, infectious diseases, cardiovascular diseases, and cancer [113,114]. To some extent, autophagy pathway dysfunction is associated with NRF2 activation. For instance, several studies have found that NRF2 can be activated through autophagy inhibition in a p62-dependent but Keap1-Cys151-independent manner, which is known as “non-canonical activation” [115,116,117,118]. Autophagy dysfunction has been shown to induce the accumulation of p62, a selective autophagy adaptor, which leads to the sequestration and loss of function of numerous binding partners, including KEAP1 [115,119,120]. p62 interacts directly with KEAP1 through its KEAP1-interacting region (KIR), which contains a DPSTGE motif similar to the ETGE motif in NRF2 for KEAP1 binding [117,121]. The KEAP1 sequestration by p62 stabilizes NRF2, which can initiate the transcription of target genes, including *p62*, creating a positive feedback loop and prolonging NRF2 activation [119]. However, the excessive accumulation of p62 induces sustained NRF2 activation, which facilitates the formation and development of tumors [122]. Deletion of *p62* consistently inhibits NRF2 activation and arsenic-induced malignant transformation of human keratinocytes [123]. The relationship between the non-canonical activation of NRF2 and carcinogenesis must be thoroughly investigated to identify a potential target for cancer treatment or prevention.

### 3.2. Dual Roles of NRF2 in Tumor

NRF2 has traditionally been considered a tumor suppressor since the NRF2–KEAP1 signaling pathway is an essential cell protection mechanism that can defend against oxidative/electrophilic stresses and promote cell survival. The activation of the NRF2 pathway induced by natural compounds is an effective chemoprevention strategy [124]. Moreover, NRF2-deficient mice are more susceptible to develop cancer, and NRF2 deficiency is associated with cancer metastasis [125,126,127,128].

Transient activation of NRF2 during stress is beneficial to normal cells, whereas hyperactivation of NRF2 facilitates the survival of normal as well as malignant cells. Recent evidence suggests that the “dark” side of NRF2 may be mediated by excessive accumulation of p21 and p62 via disruption of NRF2–KEAP1 interactions [115,129]. In addition, NRF2 can exert a significant action of chemoresistance, inhibiting drug accumulation in cancer cells, and thereby contributing to survival of cancer cells. Considering the pro-tumorigenic effect of NRF2 in cancer cells, pharmacological suppression of the NRF2 pathway will emerge as a promising area of cancer research. Several groups have identified many NRF2 pharmacological inhibitors, such as brusatol, halofuginone, luteolin, and procyanidin [130,131,132,133]. However, there is currently no FDA-approved drug to suppress NRF2 activation. Therefore, extensive research is required to identify drugs that can prevent and treat cancer.

## 4. Crosstalk between the STAT3 and NRF2 Signaling Pathways in the Tumor Microenvironment

Interestingly, the STAT3 and NRF2 signaling pathways can interact with each other (Figure 2), which undoubtedly increases the complexity of their signal transduction and the diversity of drug treatment targets. There is increasing evidence that STAT3 and NRF2 have synergistic effects in cancer cells [134,135]. Wu et al. found that IL-6 secreted by pancreatic stellate cell (PSC)-induced EMT phenotypes and gene expression in Panc-1 cells by activating STAT3, which in turn induced the expression of NRF2 and its target genes to mediate EMT [36]. EMT is a process where epithelial cells lose their cell–cell adhesion and apical-basolateral polarity and obtain mesenchymal features [136,137]. Numerous studies indicate that several metastatic cancers are caused by IL-6-induced EMT events [136,137,138]. Results from Wu et al. show that PSC-secreted IL-6 binds to its receptor and activates JAK/STAT3 signaling, which then triggers intracellular NRF2 signaling and its downstream EMT-related transcription factors to drive the expression of EMT-related marker genes, thereby inducing EMT in Panc-1 cells [36]. This study showed that the IL-6/STAT3/NRF2 signaling pathway might play a role in the progression of pancreatic ductal adenocarcinoma (PDAC) [36]. Another study found that the expression levels of both STAT3 and NRF2 were increased in HT-29 colon cancer cells, but when treated with the combination of 5-fluorouracil (5-FU) and stattic, the level of NRF2 decreased after the reduction of STAT3 expression [35]. It may be assumed that the effect of 5-FU may inhibit STAT3 and NRF2 signal transduction by blocking IL-6. The specific mechanism still needs a great quantity of research.

Moreover, in osteosarcoma cells, overactivation of STAT3/NRF2 signaling can lead to cisplatin resistance by increasing glutathione peroxidase 4 (GPX4) activity, thereby suppressing ferroptosis [135]. However, when BP-1-102, a STAT3 inhibitor, was used, the expression levels of NRF2 and GPX4 were strikingly decreased, which reactivated ferroptosis and enhanced the sensitivity of osteosarcoma cells to cisplatin [135]. 

Furthermore, both Nrf2 and STAT3 are overexpressed in breast cancer, especially in basal-like breast cancer (BLBC). Kim et al. found that NRF2 can form a stable complex with Y705 phosphorylated dimeric form of STAT3, which may accelerate the progression of breast cancer by inducing IL-23A expression [139]. IL-23A is significantly overexpressed in almost half of BLBC patients. It is worth noting that the survival rate of breast cancer patients with high levels of *IL-23A* mRNA is worse than that of patients with no or low expression of *IL-23A* mRNA [139]. IL-23 is a common proinflammatory cytokine which mainly exists in activated macrophages, dendritic cells, and keratinocytes in healthy skin [140]. However, recent studies have shown that IL-23 is also involved in tumor growth and metastasis by directly binding to the IL-23 receptor, which is expressed in a variety of in inflammation-related malignant tumors, including breast cancer [141]. The STAT3–NRF2 complex located in the nucleus where it binds to the promoter region of the *IL-23A* gene and induces its transcription. The protein products of IL-23A can bind to their receptors in BLBC cells in an autocrine manner, which will amplify the intracellular signals for breast cancer cell proliferation, migration, metastasis, etc. [139]. In view of this, the STAT3/NRF2-IL-23A axis can emphasize the importance of subtype-specific molecular pathways, which can be a potential therapeutic target.

## 5. Conclusions

STAT3, as an essential transcription factor, regulates the expression of a great quantity of genes and participates in many physiological processes, including cell growth, apoptosis, differentiation, inflammation, immunity, and angiogenesis. In normal cells, activation and inactivation of STAT3 are highly regulated, whereas, in tumor cells, STAT3 is typically overactive. Persistent STAT3 signaling can directly promote tumorigenesis by facilitating cell proliferation and inhibiting apoptosis through the upregulation of genes encoding apoptosis inhibitors (Bcl-2, Bcl-xl, Mcl-1) and cell cycle regulators (cyclins D1/D2, c-Myc) [56,142]. Moreover, in the tumor microenvironment, the endogenous negative regulators of the STAT3 signaling pathway are downregulated, resulting in enhanced proliferation and malignancy of tumor cells [54,55]. Therefore, STAT3 signaling is a viable target for cancer therapy, and the use of its inhibitors may impede the progression of cancer. However, under certain circumstances, STAT3 can act as a tumor suppressor in various tumors. This means that treatments based on STAT3 modulators should take into account the dual roles of this transcription factor and be tailored to specific tumor types.

Similar to STAT3, NRF2 is a prominent transcription factor with dual roles. Stress-induced transient activation of NRF2 is beneficial to health, whereas sustained NRF2 activation has detrimental effects. NRF2 has been considered a tumor suppressor because the NRF2–KEAP1 signaling pathway is a major cytoprotective mechanism that can defend against oxidative/electrophilic stresses and promote cell survival. However, excessive activation of NRF2 facilitates the survival of both normal and malignant cells. Recent evidence has revealed the “dark” side of NRF2, which may be mediated by the activation of a non-canonical pathway [115,116,117,118].

In addition, there is increasing evidence that the STAT3 and NRF2 signaling pathways can interact, thereby enhancing the complexity of their signal transduction. Recent research indicates that the STAT3/NRF2 signaling pathway contributes to cancer progression [35,36,134,135]. Gao et al. found that remote limb ischemic postconditioning (RIPostC) attenuated apoptosis and protected mice from myocardial ischemia/reperfusion (IR) injury, possibly by activating the JAK/STAT3-mediated NRF2-antioxidant signaling pathway [143]. This raises the question of whether the STAT3/NRF2 signaling pathway has an antioxidant effect on tumor cells by protecting them from oxidative stress damage and promoting their survival. Another study indicates that NRF2 promotes carcinogenesis in nickel-transformed cells by suppressing apoptosis and promoting autophagy via STAT3 signaling [144]. The specific regulatory mechanisms underlying STAT3 and NRF2 signaling need to be studied further under different pathological conditions.

To conclude, the signal transduction and functions of STAT3 and NRF2 are extremely intricate under physiological conditions as well as pathological conditions, particularly in tumors. Because crosstalk between STAT3 and NRF2 signaling can occur in various tumors, the specific mechanisms and functions must be determined to better guide clinical medication and new drug development.

## Data Availability

Not applicable.
